# The Oxidative Stress in Epilepsy—Focus on Melatonin

**DOI:** 10.3390/ijms252312943

**Published:** 2024-12-02

**Authors:** Maciej Kamieniak, Kamil Kośmider, Barbara Miziak, Stanisław J. Czuczwar

**Affiliations:** Department of Pathophysiology, Medical University of Lublin, 20-090 Lublin, Poland; mkamieniak70@gmail.com (M.K.); kamilkosmider96@gmail.com (K.K.); barbara.miziak@umlub.pl (B.M.)

**Keywords:** epilepsy, seizures, oxidative stress, antioxidants, free radicals, melatonin

## Abstract

Oxidative stress develops when there is an excess of oxidants leading to molecular and cellular damage. Seizure activity leads to oxidative stress and the resulting increased lipid peroxidation. Generally, antiseizure medications reduce oxidative stress, although the data on levetiracetam are ambiguous. Exogenous antioxidants (vitamin E, resveratrol, hesperidin, and curcumin) have been documented to exert an anticonvulsant effect in animal models of seizures and some recent clinical data point to curcumin as an affective adjuvant for the therapy of pediatric intractable epilepsy. Melatonin is an antioxidant with an ability to attenuate seizure activity induced by various convulsants in rodents. Its clinical effectiveness has been also confirmed in a number of clinical studies. Experimental studies point to a possibility that endogenous melatonin may possess proconvulsive activity. Moreover, some scarce clinical data seem to express this view; however, a limited number of patients were included. The anticonvulsant activity of exogenous melatonin may involve GABA-mediated inhibition, while endogenous melatonin may act as a proconvulsant due to a decrease in the brain dopaminergic transmission. Antioxidants, including melatonin, may be considered as adjuvants in the therapy of epilepsy and melatonin, in addition, in patients with epilepsy suffering from sleep disorders.

## 1. Introduction

Epilepsy is one of the most common neurological disorders, whose incidence is estimated to be around 74.5 million cases worldwide, with up to 12.5 million cases in developed countries [1]. Generally, the term epilepsy, derived from the Greek verb “epilambanein” (to seize, possess, or afflict) [2], refers to the increased susceptibility to recurrent seizures. Its causes are still unknown in a significant number of cases in high-income countries (5 out of 10 cases are idiopathic [3]), and, when they are clear, the most common ones are brain injury, stroke, and brain tumor, and can also be based on congenital and acquired defects of the nervous system during pregnancy and delivery [4]. Moreover, with all this differentiation, it is thought that, in most cases, the genetic basis plays an important role, directly, when specific mutations determine the occurrence of the disease and, indirectly, when numerous factors in combination with non-genetic ones lead to an increased susceptibility to seizures [5]. The excessive consumption of alcohol may also lead to epilepsy [6].

## 2. Search Strategy and Selection Criteria

The search for relevant articles was carried out in the following databases: PubMed, Google Scholar, and Web of Science. The keywords were: epilepsy (seizures) or antiseizure medications (antiepileptic drugs), combined with oxidative stress or antioxidants (melatonin) or reactive oxygen (nitrogen) species. Apart from recent papers, older publications were also considered, with some being definitely of historical value. Generally, the time frame was set from 1985.

## 3. Definition and Classification of Epilepsy

Supernatural concepts of epileptic etiology persisted up to the 5th century BC, until Hippocrates changed the way people looked at epilepsy, having seen the causes of disease inside the brain [2,7]. Unfortunately, this scientific vision had an extremely small impact on the common perspective on epilepsy, and, in the 17th century, it scarcely returned to favor. We had to wait until the 19th century when Jackson proposed the electrical theory of epilepsy [8]. The following years led us to an even deeper understanding of the essence of epilepsy, not as a specific disease but as a diverse group of disorders that, together, resulted in the abnormally increased predisposition to seizures.

In 2005, the International League Against Epilepsy (ILAE) and the International Bureau for Epilepsy (IBE) developed a common definition of both epileptic seizures and epilepsy. Thus, due to the ILAE and IBE, epileptic seizure is defined as a “transient occurrence of signs and/or symptoms due to abnormal excessive or synchronous neuronal activity in the brain” [9]. Epilepsy is defined as a “disorder of the brain characterized by an enduring predisposition to generate epileptic seizures and by the neurobiological, cognitive, psychological, and social consequences of this condition”, and, furthermore, “the definition of epilepsy requires the occurrence of at least one epileptic seizure” [9]. In order to complete the definition, in 2014, the ILAE issued the report creating a practical clinical definition of epilepsy. According to this, epilepsy can be found through any of the three following conditions: (1) at least two unprovoked (or reflex) seizures occurring >24 h apart, (2) one unprovoked (or reflex) seizure and a probability of further seizures similar to the general recurrence risk (at least 60%) after two unprovoked seizures, occurring over the next 10 years, and (3) a diagnosis of an epilepsy syndrome [10]. It is also worth noting that the previous definitions did not assume the possibility of outgrowing the disease, so, in the following part of the report from 2014, it is assumed that epilepsy can be considered to be resolved “for individuals who had an age-dependent epilepsy syndrome but are now past the applicable age or those who have remained seizure-free for the last 10 years, with no seizure medicines for the last 5 years” [10].

Epileptic seizures are very diverse, and the careful classification and systematization of such seizures are required. One the most recent is the New Classification of Seizures published by the ILAE in 2017 [11]. Generally, epileptic seizures can be divided into focal (formerly also called partial) seizures, which arise in one hemisphere of the brain, and generalized seizures, which arise in both hemispheres. In addition, the seizures of unknown onset stand out. Focal seizures are classified depending on whether the awareness is intact or impaired. And, further, focal seizures can be divided into motor (with subgroups automatisms, atonic, clonic, epileptic spasms, hyperkinetic, myoclonic, and tonic), non-motor (with subgroups autonomic, behavior arrest, cognitive, emotional, and sensory), and focal to bilateral tonic–clonic. Generalized seizures are basically divided into two groups, which are motor (tonic–clonic, clonic, tonic, myoclonic, myoclonic–tonic–clonic, myoclonic–atonic, atonic, and epileptic spasms) and non-motor/absence (typical, atypical, myoclonic, and eyelid myoclonia).

## 4. Neurobiology of Epilepsy

The neurobiological basis of epilepsy is synchronized neuronal discharges, while, normally, the brain’s electrical activity is nonsynchronous. These discharges relate to a specific group of neurons, usually in the cerebral cortex, which are called an epileptic focus. They can then spread to other parts of the brain resulting in symptoms of epilepsy such as abnormal movements and even sensations and thoughts [12].

In general, convulsive seizures result from the improper conduction of nerve impulses. They can be caused by the excessively frequent overstimulation of neurons and the excessively widespread spread of these stimuli. Under physiological conditions, when a stimulus neuron is unloaded, it is completely resistant to action potentials for some time (due to hyperpolarization). Moreover, epilepsy is characterized by a lowered neuronal excitability threshold [13].

Antiseizure medications (ASMs) exhibit a number of crucial activities. The three most important ones will be briefly discussed here, and these are (1) the effects on voltage-modulated ionic channels (mainly sodium), (2) the enhanced inhibitory transmission, most of all, GABA-ergic, and (3) the inhibitory effect on excitatory, mainly glutamatergic, transmission [14].

The first of these is the effect on voltage-dependent sodium channels. During stimulation, they open, causing rapid depolarization, which further leads to the release of neurotransmitters at the end of the axon, and then they close quite quickly. Another stimulation is possible only after reopening the channels. Some ASMs prolong the closure time of these channels, but act primarily on the channels depending on their condition, showing a high specificity for abnormal depolarizations than for normal discharges [15,16]. Apart from the inhibition of both sodium and calcium channels, ASMs may also activate potassium channels favoring hyperpolarization, which reduces the excitability of nerve fibers [17,18].

Excessive excitations are prevented by inhibitory neurons in which the main messenger is gamma-aminobutyric acid (GABA). The different ASMs here work in three different ways to enhance the effect of GABA. These activities include the following: (1) the activation of GABAA receptors, i.e., chloride channels, (2) increasing the GABA release, and (3) reducing its degradation [19].

As the excessive activation of N-methyl-D-aspartate (NMDA) receptors by glutamate and glycine is seen as one of the neurobiological causes of epilepsy, it is not surprising that the inhibition of the glutamatergic system is one of the ways in which ASMs work [20].

Other goals of ASMs are the glutamatergic α-amino-3-hydroxy-5-methyl-4- isoxazolepropionic acid (AMPA) receptors and, for example, synaptic vesicle glycoprotein 2A, all discussed in the description of individual drugs. In addition, the effects of ASMs on the mechanisms of oxidative stress (OS) in the course of the disease are investigated, which is the subject of this review and will be further discussed in detail and then more deeply explored in the description of individual drugs.

## 5. Oxidative Stress and Free Radicals

OS “is an imbalance between oxidants and antioxidants in favor of oxidants, leading to a disruption of redox signaling and control and/or molecular damage” [21]. Reactive oxygen species (ROS), as well as reactive nitrogen species (RNS), are oxidants found in living organisms. They form a group of molecules that are constantly produced and catabolized during the course of aerobic metabolism, as the result of the ATP (adenosine triphosphate) production by the mitochondria and/or in the wake of the exposure to external factors [22,23,24]. Both among ROS and RNS, we can distinguish free radicals as well as non-radical forms. Radical forms are characterized by at least one unpaired electron. These forms are created in three ways: (1) by breaking the chemical bond, (2) by splitting the radical, so that a different radical is formed, and (3) as a result of the redox reaction [25,26]. Non-radical ROS/RNS include the following: hydrogen peroxide (H_2_O_2_), ozone (O_3_), hypochlorous acid (HOCl), lipid peroxides (LOOH), and others. Free radicals are much more reactive than non-radical forms; however, non-radical forms can be easily transformed into radical forms [22,27]. The ROS/RNS functions in cellular signaling were discovered in the 1990s [28]. Therefore, it was considered that there is an adequate level of ROS/RNS in the organism that is optimal for maintaining homeostasis. Decreasing or increasing this level leads to disorders [29,30]. An excess of ROS and/or RNS can lead to the irreversible damage of proteins, lipids, or DNA [23]. These changes occur during the aging process [31], in chronic diseases [32], or in the course of metabolic syndrome [33].

As already mentioned, ROS/RNS are produced in the processes of oxygen metabolism. Endogenous ROS/RNS may also arise as a result of the activation of immune cells, inflammatory processes, damage caused by hypoxia, mental stress, cancer, and aging processes. The source of exogenous ROS/RNS can be air and water pollutants, alcohol, heavy metals, cigarette smoke, UV radiation, pesticides, heavy metals, or certain drugs. These factors are transformed in ROS/RNS or they induce the production of oxidants [34].

## 6. Antioxidants

Antioxidants protect against an excess of ROS/RNS. They may be divided into enzymatic and nonenzymatic ones. Enzymatic antioxidants include superoxide dismutase, catalase, glutathion peroxydase, thioredoxin reductase, and coenzyme Q10.

Superoxide dismutase (SOD) catalyzes the superoxide radical dismutase reaction. There are three types of SOD. The first is copper-zinc SOD (CuZnSOD, SOD1), which is a homodimer found in the cytoplasm and in peroxisomes. It is also placed in small quantities in the mitochondrial membranes of the secretory vesicles, endoplasmic reticulum (ER), Golgi apparatus, and nuclear envelope [35,36]. In the mitochondrial matrix, an SOD manganese homotetramer (MnSOD, SOD2) is located [37]. Extracellular SOD (ECSOD, SOD3) is located in the extracellular space. The structure of SOD3 is similar to SOD1, since it has both copper and zinc in its active site; however, it is secreted outside the cell [38,39].

Catalase (CAT) decomposes hydrogen peroxide. It may also act as a peroxidase by catalyzing the oxidation of H donors (e.g., methanol or ethanol) by H_2_O_2_. CAT, together with SOD2, has an important role in the adaptive response to increasing concentrations of oxidants in the mitochondria. CAT allows the cell to acquire a higher tolerance to oxidative stress [40]. In addition to mitochondria, it is also found in peroxisomes, as well as in the cytoplasm [41].

Glutathione peroxidase (GPx) has a key role in the reduction of H_2_O_2_. It also reduces the large-molecule lipid peroxides and products of lipoxygenase-catalyzed reactions. There are the cytosolic form of GPx, GPx connected with phospholipid membranes, gastrointestinal GPx, located in the cytosol of cells that form tissues of the gastrointestinal tract, and extracellular GPx. These enzymes are composed of four identical subunits, in the center of which there is selenocysteine (Sec). Sec is crucial for enzyme activity because, during the reduction of peroxides, GPx attaches two electrons, forming Se-OH. These electrons are then transferred to glutathione (GSH), which is necessary for a GPx catalyzed reduction. This is how H_2_O_2_ or the organic peroxides ROOH are reduced [42].

Another enzyme involved in processes aimed at the reduction of oxidants is selenoflavoprotein thioredoxin reductase (TrxR). It creates a thioredoxin system along with thioredoxin (Trx) and NADPH. It has been shown that the thioredoxin system can reduce GPx in human plasma, so that the GPx enzyme can fulfill its function, even if there is not enough glutathione [43].

Some of the endogenous antioxidants are used as markers of OS. These include CAT, SOD, Trx, and GPx. The GSH level is also analyzed [44].

Among the exogenous antioxidants, it is worth noting the likes of resveratrol, vitamin E, or curcumin, which may also have a positive effect in the treatment of epilepsy.

Resveratrol is a natural chemical compound belonging to the group of polyphenols and exhibits potent antioxidant properties. It is found in various plants, particularly in grape skins, berries, peanuts, and red wine [45]. Its antioxidative properties have been extensively studied in the context of potential applications in epilepsy treatment [46,47]. Beyond its putative anticonvulsant effects [48], resveratrol demonstrates neuroprotective properties, making it a promising adjunct in epilepsy therapy [49,50]. Rat models have demonstrated the efficacy of this compound in suppressing seizures, presumably through reducing the glutamate-induced excitotoxicity and enhancing GABAergic inhibition [51,52].

Vitamin E is a group of fat-soluble compounds with antioxidant properties [53]. Its primary role is to protect cells from oxidative damage caused by free radicals, which may also have applications in epilepsy treatment [54,55,56,57]. Studies have highlighted the neuroprotective capabilities of vitamin E in epilepsy, including the potential preservation of higher cognitive functions such as learning and memory in temporal lobe epilepsy [58]. The proposed mechanisms of action include the attenuation of energy metabolite depletion, which, in turn, mitigates oxidative stress [59].

Curcumin is an active compound derived from the root of turmeric (Curcuma longa), a widely used spice and medicinal plant with numerous health-promoting effects, including anti-inflammatory and antioxidant properties [60,61]. The exact mechanisms by which curcumin exerts its effects in epilepsy remain unclear [62]. Nevertheless, several animal models have demonstrated significant reductions in seizure activity following its administration [63,64,65]. The clinical application of curcumin may be constrained by its poor bioavailability and limited penetration into brain tissues [66]. Efforts to enhance its bioavailability for the central nervous system are ongoing, with promising results reported [67,68].

## 7. Oxidative Stress in Epilepsy

CNS consumes 20% of inhaled oxygen, with an unladen weight of only 2% of the total body weight [69]. The CNS has a high ATP demand, which is used to maintain the state of depolarization, as well as to transmit nerve impulses. In the CNS, the majority of ATP is produced in the mitochondria as a result of cellular respiration, which is why the nervous tissue has such a high oxygen demand [70]. During the production of ATP by mitochondria, ROS/RNS are also created. Their excess leads to OS and can cause mitochondrial damage [71]. OS is thought to have a significant impact on the pathogenesis of CNS diseases such as Parkinson’s disease, Alzheimer’s disease, Huntington’s disease, Freidrich ataxia, and amyotrophic lateral sclerosis (ALS) [72].

ROS also have a significant role in neuronal death and inflammation, which occur after epileptic seizures [73]. Regions vulnerable to neuronal death after SE include the hippocampal areas CA3 and CA1 and piriform cortex. There are also regions with a greater resistance to kainate (KA)-induced neuronal cell death, such as the dentate gyrus [74]. It has been proven in rat models that, after kainate-induced status epilepticus, there is a reduced activity of mitochondrial aconitase in hippocampal neurons, which is susceptible to inactivation by the superoxide radical (O^2−^) and is a reliable indicator of O^2−^ production. Such a condition occurs a few hours after SE (maximal inactivation 16 h after the end of SE) and during the death of hippocampal neurons [73]. Furthermore, reduced aconitase activity, as well as α-ketoglutarate dehydrogenase activity, which is also an enzyme susceptible to oxidative stress, was found after pilocarpine-induced seizures. These pathologies are widespread, including even areas distant from the seizure activity, although neuronal death has mainly occurred in the hippocampus [75]. Foregoing discoveries prove the increased production of oxidants after epileptic seizures.

OS, which arises after epileptic seizures, causes lipid peroxidation (LPO) in neurons. Furthermore, in adult rats, an increased activity of superoxide dismutase was observed as a result of the increasing ROS level [74].

The level of DNA damage by free radicals is monitored using 8-hydroxy-2-deoxyguanosine (8-OHdG), an oxidized derivative of deoxyguanosine. The ratio of 8-OHdG to 2-deoxyguanine (2-dG) is a reliable indicator of DNA oxidation in the cell [76]. The increase in this ratio is observed in adult rats after KA administration; it precedes postseizure neuronal death. This indicates that the oxidation of mitochondrial DNA may be important in the processes that lead to neuronal death after SE [73].

As already mentioned, after KA-induced SE, a higher concentration of oxidants occurs in regions vulnerable to neuronal death. This is important evidence that allows us to indicate OS as one of the causes of neuronal death. Another piece evidence that betokens the influence of OS on neuronal death is the fact that the susceptibility to neuronal death after KA-induced SE depends on the age of the individual. In the immature brain, the expression of mitochondrial uncoupling protein-2 (UCP-2) is higher than in adults. This protein prevents the increased production of ROS by reducing the membrane potential of the mitochondrial membrane, and also reduces intramitochondrial calcium, which is thought to release ROS production [77,78].

Neuronal death can cause the damage of the brain regions affected by epilepsy, as well as the exacerbation of seizures. However, antioxidants can stop seizure-induced neuronal death, as evidenced by the increased survival of hippocampal neurons in transgenic mice overexpressing mitochondrial SOD2, compared to wild-type mouse, after KA-induced seizures. It was also found that, in mice injected with kainate and manganese (III) tetrakis (4-benzoicacid)porphyrin (MnTBAP, an antioxidant enzyme), the loss of hippocampal neurons as well as the level of aconitase inactivation was decreased compared to mice not injected with MnTBAP. However, the administration of MnTBAP did not affect the occurrence of behavioral seizures after the KA administration [73]. Similar results were obtained in the rat model of SE-induced temporal lobe epilepsy, where the administration of the catalytic antioxidant MnIIITDE-2-ImP5+ inhibited the loss of hippocampal neurons and also prevented deficits in mitochondrial oxygen consumption rates and cognitive dysfunction [79].

However, the question arises on how OS affects epileptogenesis. It seems significant that epilepsy, although it occurs in all age groups, mostly affects older people. Older people have more free radicals, and the ability to remove them is reduced. It is believed that this is one of the basic processes leading to the aging process [80]. Evidence demonstrates that OS causes increased neuronal seizure susceptibility. This thesis is confirmed by the results of studies on Sod2−/+ mice, indicating that these mice have age-dependent spontaneous and handling-induced seizures. Moreover, they exhibit a greater susceptibility to KA-induced seizures and are more likely to lose the neurons in vulnerable hippocampal areas than Sod2+/+ mice. Most likely, the biggest role in increasing seizure susceptibility in Sod2−/+ mice is played by factors that increase the excitability of neurons. Sod2−/+ mice have age-dependent reductions in the glial expression of GLT-1 and GLAST glutamine transporters, which are sensitive to inactivation by ROS. These transporters maintain the glutamine concentration at an appropriate level, preventing its increase to toxic values. Most likely, damage to these transporters, caused by OS, leads to an increased neuronal excitability resulting in convulsions [81].

## 8. Oxidative Stress and Newer Antiseizure Medications

In view of all these facts, the question of how ASMs affect the level of OS seems to be both interesting and important. This turns out to be a popular research goal in recent years, the results of which for the newer drugs are summarized in this chapter.

Gabapentin (GBP) blocks presynaptic P/Q-type Ca^2+^ channels, which leads to a reduction in glutamate release and, consequently, decreased neuronal excitability [82]. Partial epilepsy with or without secondary generalization, in patients over 3 years of age, are the main indication. [83]. GBP is generally well-tolerated. The few adverse effects include an increased risk of suicidal thoughts, the loss of libido, anorgasmia, and impotence [84,85].

GBP seems to reduce OS, as many studies indicate. The administration of GBP in mice with hypoxic stress reduced malondialdehyde (MDA) and nitrite levels and also resulted in increased GSH and CAT activity [86]. Another study revealed that GBP inhibited OS, generated by pentylenetetrazol (PTZ)-induced convulsions. Decreased NO levels and OS were proven, which was particularly noticeable in the hippocampus and cortex [87]. Moreover, a significant reduction in OS by GBP and its neuroprotective effects against PTZ-induced convulsions in mice were observed. The same study showed the beneficial effect of GPB combined with hesperidin which is also known as an antioxidant agent [88]. However, some studies indicate that GBP in large concentrations (50 μg/mL or 100 μg/mL) causes OS in rat astrocyte cultures. On the other hand, lower GBP concentrations of 1 to 10 μg/mL seem to be well-tolerated by cortical astrocytes [89].

Tiagabine (TGB) selectively inhibits the glial and neuronal GABA uptake in the central nervous system by blocking the GABA transporter 1 (GAT-1) [90]. Focal and secondarily generalized seizures are the main indication. There are quite a few side effects like dizziness, confusion, or psychic disturbances (which are rare) [91]. One study from 2000 assessed the TGB (1–10 μg/mL) impact on OS induced by lipopolysaccharide (LPS) in astrocytes. A lower level of oxidants than in the control group was observed, proving the antioxidant properties of TGB [92]. However, at a higher concentration (10 μg/mL), TGB alone moderately increased the production of ROS (but not NO) [89,92]. Another study, with the use of astrocyte cultures, confirmed that TGB at high concentrations (<20 μg/mL) resulted in an elevated ROS production, proving the elevated levels of ROS production with damage to cellular structures including DNA. Up to 10 μg/mL, TGB did not evoke any DNA damage [93].

Vigabatrin (VGB) is an analogue of γ-aminobutyric acid (GABA), which irreversibly inhibits the action of GABA-transaminase (GABA-T), thus increasing the brain concentration of this inhibitory neurotransmitter. Its usage is commonly limited to infantile spasm (IS) and refractory complex partial seizures (CPSs) [94,95]. Side effects include dizziness, fatigue, drowsiness, and depression [96]. Cengiz et al. [97] evaluated the oxidation of proteins and lipids, GSH, GPx, and glutathione-transferase (GST) levels in the liver of rat fetuses of mothers that received VGB at various stages of pregnancy. The possible teratogenic effects of VGB were documented, due to its passing through the placenta and causing an OS with a defense mechanism insufficiency [97]. However, the use of VGB and TGB resulted in a more than threefold extension of the seizure latency period after the exposure to hyperbaric oxygen (HBO2), which increases the level of ROS and RNS. For further discussion, see ref. [97]. Similar effects were caused by the use of Na^+^ channel inhibitors, carbamazepine and lamotrigine, with oxcarbazepine being less effective [98].

Lamotrigine (LTG) inhibits the voltage-gated sodium channels, which, in turn, reduces the secretion of excitatory amino acids, primarily glutamate, and also stabilizes neuronal membranes [99]. LTG is used when treating partial seizures with or without secondarily generalized tonic–clonic seizures. It is also indicated in Lennox–Gastaut syndrome therapy [100]. Side effects concern mainly the nervous system, including asthenia, headache, dizziness, and hyperkinesia. A skin rash may be also observed [101]. A widely known but, in fact, relatively rare complication of treatment with LTG is Stevens–Johnson syndrome. A study conducted on rats evaluated the effect of LTG and carbamazepine on cognitive function and OS in the brain during PTZ-induced seizure activity. The study showed an LTG-dependent reduction in OS in comparison to the group treated with carbamazepine [102].

Haggag et al. [103] determined the serum level of MDA and GSH in a rat model of PTZ-kindled seizures (30 mg/kg on alternate days for 5 weeks) in order to assess the effect of LTG (20 mg/kg) on OS. Both the levels of MDA and GSH were significantly higher in PTZ-kindled rats than in the control group. LTG significantly prevented the PTZ-induced elevation of the serum concentration of MDA but not that of GSH [103]. Moreover, the newer report confirmed the beneficial effect of LTG (25 mg/kg), combined with vitamin B_12_ on the OS index in PTZ-kindled rats [104].

Oxcarbazepine (OXC) comprises blocking the voltage-dependent sodium channels and stabilizing the membranes, which is similar to carbamazepine’s mechanism of action. However, OXC activates cytochrome P450 enzymes to a lesser extent [100,105]. Partial seizures are the main indication [106]. There are some common side effects, like somnolence and fatigue, ataxia, headache and dizziness, and nausea and vomiting [106].

The OXC impact on OS remains unclear. When assessing GPx, CAT, SOD, and MDA levels in erythrocytes from patients with epilepsy, GPx and SOD were slightly elevated and there was no significant difference in the level of the remaining parameters [107]. Moreover, another study has shown a slight reduction in, among others, brain GSH in PTZ-kindled seizures in mice; however, this is not statistically significant when compared to the control group [108].

Topiramate (TPM) is a drug with a complex mechanism of action. It increases the activation of GABA_A_ receptors by GABA, blocks two types of receptors for glutamic acid, i.e., KA receptors and α-amino-3-hydroxy-5-methyl-4-isoxazolpropionic acid receptors (AMPA), and, last but not least, reduces voltage-dependent sodium currents [100]. Its use is broad as well as its range of activity, including for the treatment of drug-resistant epilepsy. TPM has quite a few side effects, which are ataxia, drowsiness, and dizziness [109]. There is evidence that TPM (1 mg/kg), given once daily for 12 weeks to mice with OS induced with hyperglycemia, lowered brain 4-hydroxy-2-trans-noneal (HNE; byproduct of LPO) and elevated the brain concentration of GSH in comparison with diabetic mice [110]. On the other hand, in PTZ-kindled mice brain, the GSH concentration was significantly reduced by a TPM (10 mg/kg) pretreatment and that of MDA was increased [108]. Topiramate at higher doses of 50–100 mg/kg was shown to reduce LPO in rat serum after PTZ injection or in the pyriform and frontal cortex following kainate (for review) [111]. Jafari et al. [112] studied the antioxidative properties of topiramate (100 mg/kg) in the testis tissue after ischemia reperfusion-induced oxidative injury in the rat model of testicular torsion/detorsion. This ASM significantly prevented reductions in GSH concentration and CAT or SOD activities and diminished the MDA level in testicular tissue [112].

Felbamate (FBM) is an NMDA receptor antagonist, which blocks its activation by glycine. One of the most serious side effects is severe aplastic anemia, which limits its use [113]. The main indications for FBM are, therefore, drug-resistant epilepsy and an adjuvant therapy in Lennox–Gastaut syndrome [114]. There are not many reports on FBM and its impact on OS in epilepsy treatment. One study conducted on PTZ-kindled mice revealed that chronic FBM and levetiracetam (see below) reduced seizures, nitric oxide, as well as nitric oxide synthase activity, peroxide levels, and MDA concentration in whole brain samples [115].

Levetiracetam (LEV) binds to synaptic vesicle glycoprotein 2A (SV2A) [116] and, thus, inhibits presynaptic calcium currents, which results in reduced synaptic activity [117]. Its effect on voltage-dependent sodium channels or on glutamate receptors is not convincing [100]. Having very few side effects, LEV is considered to be a very safe drug [118]. Its main indication is drug-resistant focal epilepsy [100].

LEV and FBM seem to reduce OS, as it was shown in mice using a PTZ-induced kindling model (mentioned above) [115]. Moreover, LEV (300 mg/kg) reduced OS (evaluated in the rat brain tissue) in combination with hydroalcoholic extracts of Ocimum sanctum L., which was assessed in the PTZ kindling model [119]. However, other studies indicate that, during treatment with this ASM, a reduced level of GSH and increased levels of MDA were observed, and, generally, OS was enhanced [120].

More data on newer ASMs and OS may be found in a recent publication by Kośmider et al. [121].

## 9. Melatonin

### 9.1. Melatonin Receptors

**Figure 1:**
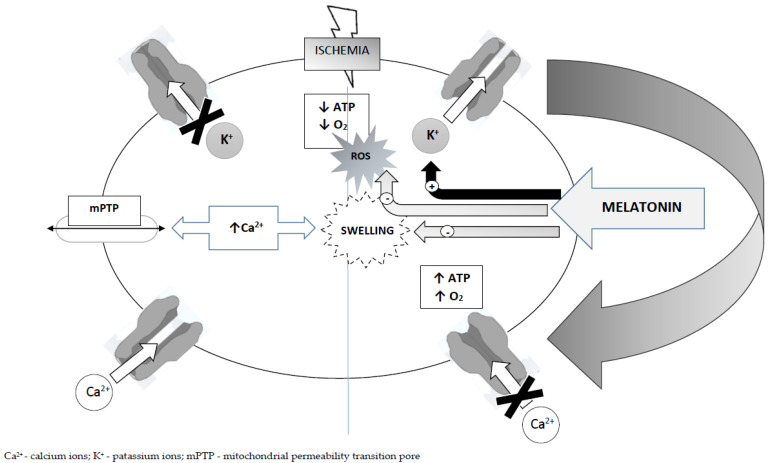
Melatonin—mechanisms of action: melatonin → ATP-sensitive potassium channels—stimulating effect; and melatonin → ROS/swelling—inhibitory effect. up arrow—increased, down arrow—decreased. For further explanations, see text.

Melatonin, as an indolamine derivative of serotonin, is synthetized in and released from the pineal gland. It may be ascribed hormonal, autacoid, or paracoid properties, and, when ingested in foodstuffs, it can be also regarded as a vitamin [122,123,124]. Due to the receptor-mediated events, melatonin may also behave as a neurotransmitter. Two receptor types (MT1 and MT2) have been described, which, in the central nervous system, are located in the mammalian neocortex, hippocampus, hypothalamus, thalamus, pituitary, and retina. Receptor signaling via high-affinity G-protein involves adenylyl cyclase, phospholipases, potassium channels, and calcium channels. Studies have also shown that melatonin accelerates conduction in ATP-sensitive potassium channels (KATP). Intracellular ATP levels physiologically regulate the opening and closing of potassium channels. Typically, fluctuations in this ATP level are negligible except in states of marked metabolic disturbance, prominent among which is the ischemic/hypoxic state seen in epileptic seizures. Hypoxia leads to the excessive consumption of ATP in metabolic processes, and, consequently, a decrease in cellular ATP levels and pH. In such a case, KATP channels are opened, followed by a shortening of the potential duration in the cell after depolarization. The consequence of such a condition is the closure of channels for Ca^2+^. This is important because excessive Ca^2+^ loading contributes to edema and induces at least apoptosis/necrosis by impairing the mitochondrial membrane, causing the activation of mPTP (mitochondrial permeability transition pore). In addition, opening KATP channels prevents the further loss of ATP [125,126,127] (Figure 1). The operation of the above mechanism is very well-demonstrated by experiments in which mice lacking the subunit of KATP channels showed a very high sensitivity to hypoxia and exhibited a reduced threshold for generalized seizures [128].

Although both the MT1 and MT2 receptors are distributed within the hippocampus, the former seems to prevail [123]. A low-affinity membrane MT3 receptor is also known, associated with calcium and calmodulin, and involved in the regulation of intraocular pressure [123]. Apart from its receptor-mediated effects, melatonin is an ROS/RNS scavenger. The indolamine is also capable of inducing the activity of antioxidative enzymes, which results in a reduction of OS [123]. There are also data indicating that melatonin may inhibit glutamate-mediated excitatory events in the striatum via NMDA glutamatergic receptors [123]. Moreover, melatonin was documented to elevate the brain concentration of GABA, increasing the GABAA receptor affinity as well [123]. However, this modulatory influence of the indolamine on the GABAA receptor complex may be dependent on the type of melatonin receptor involved [129]. According to Stewart [130], endogenous melatonin inhibits the brain dopaminergic transmission, which may result in an increase in seizure activity. Indeed, the intrahippocampal administration of melatonin antagonists produced an anticonvulsant effect (see below). It is noteworthy that the stimulation of brain dopaminergic D_2_ receptors has been documented to exert an anticonvulsant effect [131].

### 9.2. Effects of Melatonin upon Seizure Activity in Experimental Models of Seizures

Numerous data indicate that melatonin exerts an anticonvulsant activity in different seizure models. Indeed, melatonin at 50 mg/kg raised the threshold for maximal electroconvulsions in mice and this effect was prevented by bicuculline (1 mg/kg) and picrotoxin (1.5 mg/kg). At the subthreshold dose of 25 mg/kg, melatonin potentiated the protective activity of carbamazepine and phenobarbital against mouse maximal electroshock-induced convulsions, pharmacokinetic interactions being excluded. Once more, bicuculline inhibited the melatonin-induced enhancement of the protective activity of these ASMs [132]. In the same seizure model, similar results were obtained, with regard to carbamazepine, which also points to a beneficial interaction between phenytoin and melatonin [133]. According to Mevissen et al. [134], melatonin (at doses starting from 75 mg/kg) elevated the afterdischarge threshold and shortened the duration of generalized seizures in amygdala-kindled rats. As for acute intravenous PTZ-induced convulsions in mice, melatonin (at 40 and 80 mg/kg) raised the threshold for clonic seizure activity [135]. It was also effective (at 10 mg/kg) against PTZ-induced seizures in guinea pigs, in terms of seizure severity and lethality [136]. Further experiments with melatonin and some of its derivatives against acute PTZ seizures confirm their anticonvulsant efficacy in this seizure model in mice [137,138,139]. After all, melatonin (20 mg/kg/day for 15 days) lowered the brain level of MDA and raised that of the SOD vs. PTZ alone group [139]. Mosińska et al. [137] also demonstrated the anticonvulsant activity of melatonin (50 and 100 mg/kg) against threshold electroconvulsions (maximal electroshock threshold and 6 Hz tests), pointing to its low neurotoxic potential in terms of motor coordination and muscle strength. Strikingly, the melatonin receptor agonists Neu-P11 and Neu-P67 exerted no anticonvulsant activity [137].

There are also data indicating that melatonin may protect against kainate-induced seizures. For instance, when injected at 5 mg/kg prior to ip kainate (40 mg/kg), it reduced the seizure activity in mice and prevented an increase in the brain LPO. Moreover, melatonin significantly inhibited kainate-induced massive neurodegeneration in the CA3 area of the hippocampus [140]. Further, kainate (45 mg/kg)-induced convulsions were completely abolished by melatonin (20 mg/kg) in mice and this anticonvulsive effect was accompanied by its protective activity against the kainate-produced damage to cortical mtDNA and the prevention of an increase in the brain LPO in vivo [132,141]. Studies in vitro yielded similar results with regard to the effect of melatonin (1.5 mM) on LPO and damage to mtDNA [130,142]. Melatonin (60 mg/kg) was also found to be effective against kainate (30 mg/kg)-induced convulsions in mice and exerted an antioxidant effect as measured by hippocampal GSH. The aorylhydrazone derivative of melatonin, melatonin 3e, exerted protective and antioxidant effects comparable to melatonin [143]. Interestingly, luzindole (a melatonin receptor antagonist with a higher affinity at M2 receptors [144]) fully attenuated the anticonvulsant activity of both compounds. As for their antioxidant effect, only that of melatonin was completely blocked, the effect of melatonin 3e being only partially affected [143]. Mohammadi et al. [127] are of the opinion that the anticonvulsant activity of melatonin (40 and 80 mg/kg) against intravenous PTZ in mice might involve ATP-sensitive potassium channels. In fact, the melatonin-induced increase in the convulsive clonic seizure threshold was reduced by a channel blocker, glibenclamide. On the contrary, ATP-sensitive potassium channel openers (diazoxide or cromakalin) enhanced the anticonvulsant action of the indolamine [127].

The induction of status epilepticus by means of the electrical stimulation of various brain structures or the administration of convulsants (i.e., kainate or pilocarpine) leads to the occurrence of spontaneous seizures in rodents, following a silent period for the process of epileptogenesis that may last for several weeks [145]. This model was employed by Li et al. [146] for the evaluation of melatonin effects on spontaneous seizure activity in mice after intrahippocampal kainate-induced status epilepticus. Melatonin (10 mg/kg) was started three days after kainate injection and was given singly or for a week. Spontaneous seizure activity recorded with EEG indicated that melatonin in the single dose reduced the seizure frequency. After repeated injections, the indolamine decreased the number and severity of behavioral spontaneous convulsions and melatonin-pretreated mice exhibited an improvement in cognition, learning, and memory tasks. A neuroprotective effect in the hippocampus was also observed. Moreover, the pretreatment with melatonin altered the polarization status of microglia from a proinflammatory phenotype (M1) to an anti-inflammatory M2 phenotype, probably by the inverse regulation of the RhoA/ROCK signaling pathway [146].

Ramelteon (a melatonin M1 and M2 receptor agonist) was studied in two animal models—the rat rapid kindling model and the spontaneously epileptic Kcna1-null mouse model [147]. In the rapid kindling model, the agonist (given twice daily at 30 or 100 mg/kg for five days) reversed the hippocampal excitability following the kindling procedure, being ineffective on the baseline afterdischarge properties and kindling progression. In Kcna1-null mice, the agonist (200 mg/kg daily) diminished the seizure periodicity and frequency. Improved circadian rest–activity rhythms were also observed [147]. Ramelteon (3 and 10 mg/kg in repeated doses) was also tried against intrahippocampal kainate in mice. The agonist alleviated OS, restored glutathione homeostasis, reduced microglia activation, and mitigated proinflammatory phenotypic changes in hippocampal astrocytes [148]. In addition, ramelteon exerted a neuroprotective effect in the hippocampus, and decreased memory impairment and depression-like behaviors in kainate-treated mice. The beneficial effects of ramelteon were shared by melatonin at 10 mg/kg [148].

There are also scarce data showing a proconvulsive potential of melatonin, however, only derived from an in vitro study or dealing with endogenous melatonin [149,150]. At a concentration of 1 µmol (but not at 10 nM), melatonin increased the epileptiform activity recorded from rat hippocampal slices placed in a low magnesium concentration. The proconvulsive effect was only seen in daylight. It is noteworthy that luzindole (10 µmol) blocked melatonin’s proconvulsive activity [149]. In another study, the intrahippocampal administration of melatonin receptor antagonists resulted in an increased latency to pilocarpine-induced convulsions in rats during the dark phase, this effect not being observed during the light phase. The authors conclude that this may speak for the proconvulsive activity of endogenous melatonin [150].

The data on the effects of melatonin on seizure activity in animal models are shown in Table 1, Table 2 and Table 3.

### 9.3. Results from Clinical Studies

Melatonin was tried in the form of adjunctive therapy in clinical conditions (including adult and pediatric patients); however, some of them were based on a limited number of patients. For instance, melatonin (3 mg daily, 30 min before bedtime) was added to the existing ASMs in six children with intractable seizures [151]. In five of them, clinical improvement was observed in terms of seizure activity. EEG monitoring in two children revealed reduced epileptic activity during sleep [151]. In the randomized, double-blind, placebo-controlled study, quality of life was evaluated in 31 children on valproate monotherapy (16 on add-on melatonin) [152]. The results indicate that the attention, memory, and language subscales were significantly improved. Other investigated parameters, cognition, anxiety, and behavior, were also better in the group receiving melatonin [152]. A randomized controlled trial was conducted in adult patients with generalized onset motor seizures [153]. The control group of patients (N = 52) received valproate (20 mg/kg) + placebo and the test group (N = 52) received valproate + melatonin (3 mg/day). Among the evaluated parameters, the responder and seizure-free rates were significantly higher in the test group. Moreover, an improvement in quality of life was noted in the test group [153]. Another randomized controlled trial was carried out in 60 patients with idiopathic generalized tonic–clonic seizures under valproate monotherapy [154]. The patients were either on melatonin or placebo intermittently, with a two-week washout period. Melatonin proved quite effective as the mean severity score of epilepsy was reduced from 32.22 ± 9.24 to 5.58 ± 14.28 (*p* = 0.02). However, although the number of epileptic attacks was not decreased, the sleep quality was distinctly improved [154]. There are also recent data from a randomized, placebo-controlled, double-blind trial indicating that melatonin may exert a beneficial effect against infantile epileptic spasms syndrome as an add-on treatment [155]. Melatonin (3 mg daily given 0.5–1 h before bedtime) was added to a standard treatment consisting of the adrenocorticotrophic hormone and magnesium sulfate. The mean percentage spasm frequency was reduced to 66.7% but this result did not reach significance. In any case, improved sleep quality was evident in melatonin patients—regular sleep was reported in 85.7% of patients vs 42.9% in the placebo group. Melatonin adverse effects were not observed and were similar in both the placebo and tested groups [155].

Less numerous studies point to the proconvulsive potential of melatonin. For instance, this free radical scavenger (5 mg daily) was applied in six neurologically disabled children with sleep disorders, five of them also exhibiting epileptic seizures [156]. Although sleep quality was considerably improved, four children had increased seizure activity, which returned to baseline following melatonin withdrawal [156]. A case-report study describes a 21-year-old female patient (taking carmazepine at a dose of 600 mg/daily, phenytoin 200 mg/daily, and valproate 1500 mg/daily) experiencing one to three generalized tonic–clonic seizures a day [157]. Following the administration of melatonin (1.5 mg/daily), megentoencephalographic recordings revealed melatonin’s proconvulsive activity. Moreover, the patient had four brief seizures after taking melatonin at 2 p.m. [157].

## 10. Conclusions

Numerous data indicate that OS, apart from epilepsy, may be involved in the pathogenesis of various diseases of the central nervous system, including Alzheimer’s disease, Parkinson’s disease, and amyotrophic lateral sclerosis. ROS/RNS have been documented to participate in neuronal death and neuroinflammation, which play an important role in epileptogenesis [158]. Considering that seizure activity is tightly associated with increases in OS, it is thus quite clear that recurrent seizures still promote the process of epileptogenesis. A question emerges as to whether available exogenous antioxidants and/or ROS/RNS scavengers may be considered for the therapy of epilepsy and as possible antiepileptogenic compounds. The results of the experimental studies clearly indicate that such compounds can inhibit seizure activity—for example, vitamin E, hesperidin (PTZ-induced convulsions in rats and mice, respectively), dimethyl fumarate (rats), hesperidin (mice), resveratrol (rats; kainate-induced seizures), coenzyme Q10 (mice), curcumin (rats; PTZ-kindled seizures and spontaneous seizure in rats after kainate-induced status epilepticus), vitamin C (rats), sulforaphane (mice), and lipoic acid (rats; pilocarpine-induced convulsions) (for review) [158,159]. Having in mind that PTZ-induced kindled seizures may be regarded as a model of epileptogenesis [160], some exogenous antioxidants can thus possess antiepileptogenic properties.

Data on the effects of melatonin upon seizure activity are ambiguous. There are results of experimental studies indicating that indolamine may raise the convulsive threshold or potentiate the protective activity of some ASMs. In some studies, luzindole was co-administered with melatonin or melatonin agonists—such studies provide evidence that the influence of melatonin and/or its agonists on seizure activity was reduced with regard to both the anticonvulsant and proconvulsant (in vitro) effects. Whilst exogenous melatonin is rather anticonvulsant, the endogenous indolamine may exert proconvulsive activity. The former may be anticonvulsant due to its influence on melatonin receptors and/or GABA-mediated events, glutamatergic neurotransmission, and ATP-sensitive potassium channels (see above). On the contrary, the latter in an in vivo experiment was clearly proconvulsant, as its antagonists protected against pilocarpine-induced seizures but only at the dark phase.

When combined with ASMs, the indolamine potentiated the anticonvulsant activity of carbamazepine and phenobarbital, whilst the activity of phenytoin and valproate was not altered against maximal electroconvulsions in mice. Considering that bicuculline attenuated the melatonin-induced enhancement of the protective efficacy of carbamazepine and phenobarbital, a GABA-ergic involvement may be postulated. Strikingly, the indolamine significantly worsened long-term memory when either given alone or combined with both ASMs [132]. It should be accentuated that this was an acute effect of melatonin in naïve animals. Following repeated administration, melatonin or its agonist, agomelatine, were shown to improve memory (disturbed by prior interventions) in rodents [146,161].

Seizure activity may be related to an increased brain melatonin concentration, according to the circadian rhythm [130]. In such cases, melatonin in pharmacological doses, resulting in a much higher brain concentration, may prove to be particularly effective. After all, numerous clinical data indicate that the indolamine may be effective as an adjuvant against seizures unrelated to the circadian rhythm (see above). In any case, melatonin (or its agonists, for instance, agomelatine) may be applied in patients with epilepsy and accompanying sleep disorders [162,163]. Other antioxidants may also be considered as adjuvants in the management of epilepsy, with the experimental background clearly pointing to such a possibility [159]. The clear-cut clinical efficacy of curcumin in children with intractable epilepsy strongly supports this therapeutic strategy [62].

## Figures and Tables

**Table 1:** Effect of melatonin on the seizure threshold.

Melatonin Dose [mg/kg]	Administration		Convulsion Model	Animal Model	Seizure Threshold	Bibliography
	Acute	Chronic				
20/day	-	+ (for 15 days)	PTZ	Mice	↑	[139]
50	+	-	MEST	Mice	↑	[132]
50	+	-	PTZ	Mice	0	[137]
			6 Hz		↑	
			MEST		↑	
60	+	-	PTZ	Mice	↑	[138]
20/day	-	+ (for 15 days)	PTZ	Mice	↑	[139]
75 and 100	+	-	generalized seizures in amygdala-kindled model	Rats	↑	[134]
100	+	-	PTZ	Mice	↑	[137]
			6 Hz			
			MEST			

“-”—not applicable/no data; +—tested; 0—no significant changes; ↑—increased; KA—kainic acid; MEST—maximal electroshock seizure threshold; PHT—phenytoin; PTZ—pentylenetetrazol.

**Table 2:** Melatonin and seizure activity.

Melatonin Dose [mg/kg]	Administration		Convulsion Model	Animal Model	Anticonvulsant Effect		Bibliography
	Acute	Chronic			Combination with AEDs	Effect	
5	+	-	Kainate-induced (40 mg/kg)	CD2-F1 Mice	-	↑	[140]
10	+	+	Intrahippocampal kainate-induced status epilepticus	Mice	-	↑	[146]
10	+	-	PTZ	Guinea pig	-	↑	[136]
20	+	-	Kainate-induced (45 mg/kg)	Mice	-	↑	[142]
Melatonin 20 (30 min before the kainate administration)	+	-	Kainate-induced (45 mg/kg)	Mice	-	↑	[141]
Melatonin 20 (given simultaneously with kainate)						↑	
Melatonin 20 (15 min after the kainate administration)						↓	
25	+	-	MES	Mice	CBZ	↑	[132]
					PB	↑	
					VPA	0	
40	+	-	PTZ	Mice	-	↑	[135]
80	+	-	PTZ	Mice	-	↑	[135]

“-”—not applicable/no data; +—tested; 0—no significant changes; ↑—increased; ↓—decreased; CBZ—carbamazepine; MES—maximal electroshock seizure; PB—phenobarbital; PTZ—pentylenetetrazol; VPA—valproate.

**Table 3:** Melatonin and brain levels of MDA/SOD/LPO.

Melatonin Dose [mg/kg]	Administration		Brain Level of MDA/SOD/LPO	Bibliography
	Acute	Chronic		
5	+	-	↓ LPO	[140]
20	+	-	↓ LPO	[142]
20/day	-	+ (for 15 days)	↓ MDA ↑ SOD	[139]
Melatonin 20 (30 min before the kainate administration)	+	-	↑ LPO	[141]
Melatonin 20 (given simultaneously with kainate)			↓ LPO	
Melatonin 20 (15 min after the kainate administration)			↑ LPO	

“-”—not applicable/no data; +—tested; ↑—increased; ↓—decreased; LPO—lipid peroxidation products; MDA—malondialdehyde; SOD—superoxide dismutase.

## Data Availability

Not applicable.
